# Supplementary material for: Characterization of vaginal microbiota in Thai women
Source: PeerJ. 2018 Nov 21;6:e5977. doi: 10.7717/peerj.5977 (PMC6252066; doi:10.7717/peerj.5977)
Supplement: Table S3 [file peerj-06-5977-s003.docx]

**Supplementary Table 3** The bacterial genera found in vaginal samples, ranked by abundance, in the lactobacilli-dominated and non-lactobacilli dominated groups.

| **Lactobacilli-dominated group**  **%*** | |  | **Non-lactobacilli dominated group**  **%*** | |
| --- | --- | --- | --- | --- |
| *Lactobacillus* | 89.660 |  | *Gardnerella* | 40.535 |
| *Pseudomonas* | 4.664 |  | *Atopobium* | 24.791 |
| *Gardnerella* | 3.303 |  | *Lactobacillus* | 13.631 |
| *Sphingobium* | 1.582 |  | *Prevotella* | 8.268 |
| *Atopobium* | 0.390 |  | *Megasphaera* | 3.505 |
| *Bifidobacterium* | 0.286 |  | *Pseudomonas* | 3.040 |
| *Prevotella* | 0.058 |  | *Aerococcus* | 1.554 |
| *Finegoldia* | 0.028 |  | *Sneathia* | 1.051 |
| *Desulfosporosinus* | 0.008 |  | *Parvimonas* | 1.044 |
| *Ureaplasma* | 0.006 |  | *Streptococcus* | 0.912 |
| *Howardella* | 0.006 |  | *Mageeibacillus* | 0.777 |
| *Streptococcus* | 0.003 |  | *Dialister* | 0.222 |
| *Anaerococcus* | 0.002 |  | *Gemella* | 0.145 |
| *Peptostreptococcus* | 0.001 |  | *Peptoniphilus* | 0.145 |
| *Sphingomonas* | 0.001 |  | *Anaerococcus* | 0.126 |
| *Bacteroides* | 0.001 |  | *Arcanobacterium* | 0.068 |
| *Peptoniphilus* | 0.001 |  | *Ureaplasma* | 0.042 |

* The values are the average percentage abundance of the corresponding genera in each group.
